# Use of a Mixture of Polyols Based on Metasilicic Acid and Recycled PLA for Synthesis of Rigid Polyurethane Foams Susceptible to Biodegradation

**DOI:** 10.3390/ijms22010069

**Published:** 2020-12-23

**Authors:** Joanna Paciorek-Sadowska, Marcin Borowicz, Ewelina Chmiel, Jacek Lubczak

**Affiliations:** 1 Department of Polyurethane Chemistry and Technology, Institute of Materials Engineering, Kazimierz Wielki University, Chodkiewicza 30 St, 85-064 Bydgoszcz, Poland; 2 Department of Organic Chemistry, Rzeszów University of Technology, Al. Powstańców Warszawy 6, 35-959 Rzeszów, Poland; ewelinachmiel@prz.edu.pl (E.C.); jml@prz.edu.pl (J.L.)

**Keywords:** polyurethane foam, properties of foam, synthesis of polyols, metasilicic acid, PLA

## Abstract

Two polyol raw materials were obtained in the conducted research, one based on metasilicic acid (MSA), the other based on poly(lactic acid) (PLA) waste. The obtained polyols were characterized in terms of their applicability for the production of rigid polyurethane foams (RPUFs). Their basic analytical properties (hydroxyl number, acid number, elemental analysis) and physicochemical properties (density, viscosity) were determined. The assumed chemical structure of the obtained new compounds was confirmed by performing FTIR and ^1^H NMR spectroscopic tests. Formulations for the synthesis of RPUFs were developed on the basis of the obtained research results. A mixture of polyols based on MSA and PLA in a weight ratio of 1:1 was used as the polyol component in the polyurethane formulation. The reference foam in these tests was a foam that was synthesized only on the basis of MSA-polyol. The obtained RPUFs were tested for basic functional properties (apparent density, compressive strength, water absorption, thermal conductivity coefficient etc.). Susceptibility to biodegradation in soil environment was also tested. It was found that the use of mixture of polyols based on MSA and PLA positively affected the properties of the obtained foam. The polyurethane foam based on this polyol mixture showed good thermal resistance and significantly reduced flammability in comparison with the foam based MSA-polyol. Moreover, it showed higher compressive strength, lower thermal conductivity and biodegradability in soil. The results of the conducted tests confirmed that the new foam was characterized by very good performance properties. In addition, this research provides information on new waste management opportunities and fits into the doctrine of sustainable resource management offered by the circular economy.

## 1. Introduction

The increasing awareness of the destruction of the ozone layer and stringent requirements regarding the fire safety of building materials have resulted in significant changes in the production process of thermal insulation materials, resulting in, e.g., wide use of polyurethane foams [1,2]. Polyurethanes are one of the fastest growing groups of polymeric materials. They are one of the most universal, modern and safe groups in the field of plastics. Foams constitute the majority of the world’s polyurethanes production. Current trends direct the production of polyurethane materials to the use of environmentally friendly raw materials, the addition of raw materials from renewable sources and solving the problem of post-production and post-consumer waste at the design stage of the production process. Building materials (including polyurethane foams) are subject to high requirements regarding their physicochemical, mechanical and flammability properties [3].

For many years, new systems of raw materials have been developed in various research centers in order to obtain materials with properties that meet the high requirements of quality and fire safety standards. The basic raw materials used to obtain polyurethane materials include, among others polyols. The largest source of polyol raw materials are products derived from processing of crude oil. Current research and technology trends aim to replace them with raw materials of natural origin [4] or with raw materials obtained as a result of recycling (extending the “life cycle”) [5,6,7]. Such research is carried out in two aspects: replacing components from non-renewable sources with their renewable counterparts and reducing the emission of non-degradable waste into the environment as a result of introducing components that favor their biodegradation [8,9]. Such possibilities are created by the synthesis of plant-based bio-polyols consisting of the processing of vegetable oils for the production of polyol raw materials. The introduction of vegetable oils as raw materials for industrial production is very important both from an ecological and economic point of view. The literature provides many examples of polyurethane foams modified with bio-polyols obtained from various vegetable oils, including from rapeseed, soybean, sunflower, palm and linseed oils [4,10,11,12]. On the other hand, the use of recycled polyols is also advantageous from the point of view of saving petrochemical raw materials. In the case of using waste based on natural resources (e.g., bio-polymers), it may also facilitate the biodegradation of the obtained materials. The addition of recycled material also has great importance from the point of view of the circular economy and the implementation of the sustainable development doctrine [5,6].

Glycolysis of poly(ethylene terephthalate) (PET) with amines or alkanolamines in the presence of KOH is very important among many methods of obtaining polyol raw materials as a result of chemical recycling of waste products [13,14]. PET glycolysis processes and their modifications to obtain polyols for polyurethanes have been carried out on an industrial scale for several years. Using them for the production of rigid polyurethane foams is currently one of the economically viable solutions. Polyester polyols obtained by glycolysis of poly(ethylene terephthalate) are used due to the possibility of obtaining products with different molecular weights and physical properties depending on the glycol content, stoichiometric ratio of reactants and reaction conditions [15]. Polyurethane foam waste can also be a valuable raw material in the technology of obtaining polyol raw materials. The main purpose of the chemical recycling of polyurethanes is to obtain a liquid product with properties enabling its use in a polyurethane formulation. Many methods of chemical recycling of PUR have been described in the literature (e.g., aminolysis, acidolysis, hydrolysis) [16]. However, one of the cheapest methods of obtaining liquid polyols from polyurethane foam waste is glycolysis reaction. This process has been modified many times over the years in order to solve various technological problems. Complications in the process result from the type of polyurethane material, which is glycolyzed. Therefore, these methods differ in the type of waste, glycol, PUR:glycol ratio, type and amount of catalyst, temperature and reaction time. It was found, on the basis of many studies, that PUR foams made of recycled polyols exhibit excellent strength and thermal insulation properties [17].

The performance requirements for polyurethane foams are closely related to the directions of their application. They can be modified in a wide range, through the appropriate selection of raw materials and methods of their production. The major disadvantages of classic polyurethane foams are their high flammability and low thermal resistance. Polyurethane foams burn rapidly releasing large amounts of heat. A lot of toxic gases and smoke are also emitted during the combustion. Moreover, the maximum temperature at which they can be used without significantly deteriorating their properties is 110 °C. The search for thermal-resistant, flame-retardant polyurethane materials is currently a very important research challenge. Due to restrictive legal regulations regarding environmental protection, flame retardants that do not contain halogens are being sought. Halogen flame retardants used on a large scale until recently showed a negative impact on the environment (toxic properties) [18,19].

Polyols with silicon atoms embedded in their structure [20] are a rare subject of research in terms of their use in the production of polyurethane foams. More often, we can find information on the introduction of these atoms with inorganic fillers into polyurethane compositions. Talc, mica, montmorillonite have a hydrophilic character. These fillers additionally require intercalation with molecules of amino acids, ammonium or phosphonium salts to make them compatible with the polyurethanes. Otherwise, the phase segregation occurs, reducing the performance properties of the composite. The addition of silica nanoparticles to the polyurethane foam leads us to obtain materials with improved mechanical and thermal properties and increased fire resistance, although it hinders pore formation process [21,22]. Increasingly, we can find information about silsesquioxanes used as modifiers of thermal resistance of polyurethanes. These compounds contain hydroxyl groups as end groups and can react with isocyanate groups of urethane prepolymers, giving polyurethanes with increased thermal resistance [23,24]. The search for new polyol raw materials has led to the use of metasilicic acid (MSA) to obtain polyetherols containing silicon heteroatoms. Such polyols can be successfully used to obtain rigid polyurethane foams with favorable functional properties, e.g., high thermal resistance, lower flammability or increased mechanical strength [25].

The aim of the article was to obtain two polyols—polyetherol based on MSA and the product of glycolysis of waste from poly(lactic acid) [PLA], then using them in the mixture for the production of rigid polyurethane foams (RPUFs) and checking the influence of the mixture on the performance properties of the obtained materials

## 2. Results and Discussion

### 2.1. Properties of Synthesized Polyols

The synthesized polyols were subjected to analytical, physico-chemical tests to determine their basic properties and spectroscopic tests to confirm their assumed chemical structure. The properties of MSA-based polyol and PLA-based eco-polyol are shown in Table 1.

The color and smell of the obtained polyols strongly depended on their synthesis processes and the raw materials used for them. In the case of the eco-polyol based on PLA waste, a light-yellow liquid without any characteristic smell was obtained. This color of the polyol raw material resulted from the use of light-colored waste (mainly from white to yellow) and a relatively large amount of ethylene glycol (MEG), which also led to the lightening of obtained color. The lack of the smell of eco-polyol was also a desirable feature. This proved the correctness of the synthesis process and the absence of initial raw materials (e.g., MEG) or by-products (e.g., products of thermal decomposition of PLA). The MSA-based polyol was a brown liquid that also did not have any smell. The reason for this color was that the reaction was carried out at a high temperature for 14 h.

The densities of the obtained polyols were 1.250 g/cm^3^ for the MSA-based polyol and 1.188 g/cm^3^ for the PLA-based eco-polyol, respectively. During the synthesis of the first polyol, a gradual decrease in density was observed in relation to the initial raw material, which was metasilicic acid (bulk density of MSA—2.4 g/cm^3^). Adding subsequent segments to the MSA molecule derived from GL and EC resulted in obtaining a liquid product with a lower density than MSA. The synthesis of the PLA-based eco-polyol also resulted in a polyol raw material with a lower density than the waste used for reaction. The PLA used for the synthesis had a density in the range of 1.25–1.27 g/cm^3^, while the ethylene glycol used as a glycolyzing agent had a density of 1.11 g/cm^3^. This meant that the use of PLA:MEG mass ratio of 1:1 resulted in obtaining a polyol with an intermediate density between PLA waste density and MEG density. In the case of viscosity tests of the synthesized polyols, a very large difference in this parameter was noticed. The MSA-based polyol had a viscosity of 33,442 mPa·s. Such high value of this parameter resulted from the highly branched structure of the obtained compound and an increase in its molecular weight. On the other hand, the PLA-based polyol was a product with very low viscosity (210 mPa·s). The use of a low-viscosity glycolyzing agent (16 mPa·s at 25 °C) and a very high mass ratio of PLA:MEG led to the obtaining a product with oligomeric (no longer polymeric) chains. Shorter chains were more mobile, which resulted in a lower viscosity of the eco-polyol raw material. Too low and too high a value of viscosity is an undesirable phenomenon from an economic point of view. In industrial practice, raw materials with high viscosities require the use of specialized pumps or require additional heating, which allows us to reduce this parameter for optimal processing. This has a direct impact on the increase in production costs. The measurement of the pH showed that the value of this parameter for MSA-based polyol was 5.0, while for PLA-based polyol it was 6.0. This meant that the obtained products were acidic or slightly acidic. In the case of the eco-polyol, this pH was the result of the release of carboxyl groups at the end of the chains during the glycolysis reaction. In turn, the pH of MSA-based polyol was caused by the substitution of only one hydrogen atom in MSA with a hydroxyalkyl group. The second Si-OH group was then free, which decreased the pH of the polyol. 

The hydroxyl number (HN) is a parameter that indirectly determines the amount of hydroxyl groups in a polyol molecule. The higher the HN, the greater the amount of free hydroxyl groups in the compound [9]. High values of this parameter were noted for both polyol raw materials. The HNs were 807 mg KOH/g for MSA-based polyol and 491 mg KOH/g for PLA-based polyol, respectively. The high HN value for the MSA-based polyol was due to the branched structure of this raw material containing four free primary hydroxyl groups in one molecule. In turn, the HN of eco-polyol resulted mainly from the use of an equal amount of ethylene glycol in relation to the PLA waste. A large amount of the glycolyzing agent led to obtaining more shorter chains of lactic acid oligomer, while increasing HN of this product [6]. The acid numbers (AN) of the obtained polyols were 4.2 mg KOH/g for MSA-based polyol and 2 mg KOH g for PLA-based polyol, respectively. This parameter means the amount of free carboxyl groups contained in the analyzed compound. In the case of the PLA-based polyol, the increased amount of free COOH groups resulted from the glycolysis reaction. Each PLA macromolecule was ended with this group. In this case, there was not an esterification reaction of free carboxyl groups with ethylene glycol, because the used catalyst (zinc stearate) promoted the transesterification reaction. In turn, the AN of the MSA-based polyol resulted from the presence of a small amount of unreacted Si-OH bonds and a small amount of poly(silicic acid) (9.5 wt.%). The polyols obtained in this research were subjected to gel permeation chromatography (GPC). This test was aimed at determining the number average molecular weight, the weight average molecular weight, and the polydispersity. GPC analysis of the tested samples showed that the number average molecular weight and the weight average molecular weight of PLA-polyol was at similar level (309 and 351 g/mol, respectively). However, significant differences were observed in the case of MSA-polyol (314 and 899 g/mol, respectively). An exact description of the reason for this difference is presented in the Supplementary Materials of this article (Table S1, Table S2, Table S3 and Table S4 and Figure S1). An important parameter of the obtained polyols is their polydispersity. This is the ratio of the weight average molecular weight to the number average molecular weight. The polydispersity of the obtained polyols was 1.14 for eco-polyol based on PLA waste and 2.86 for MSA-based polyol. The low value of this parameter is very advantageous from the point of view of using these raw materials for the synthesis of polyurethane materials [26]. In turn, The high value of polydispersion (above 1.8) could significantly reduce or exclude the use of these raw materials in the production of polyurethanes, which should have an ordered structure and have beneficial properties [27,28].

The synthesized polyols were also analyzed for their elemental composition. This study was particularly important in the case of the analysis of the share of individual elements in the compound. Knowing the elemental composition of foam, it is possible to calculate the theoretical oxygen demand (TOD), which is necessary for susceptibility to biodegradation test. In turn, this parameter is important when calculating the biodegradation degree of the tested material. The obtained results confirmed the assumed proportion of individual elements in the synthesized polyols.

**Figure 1:**
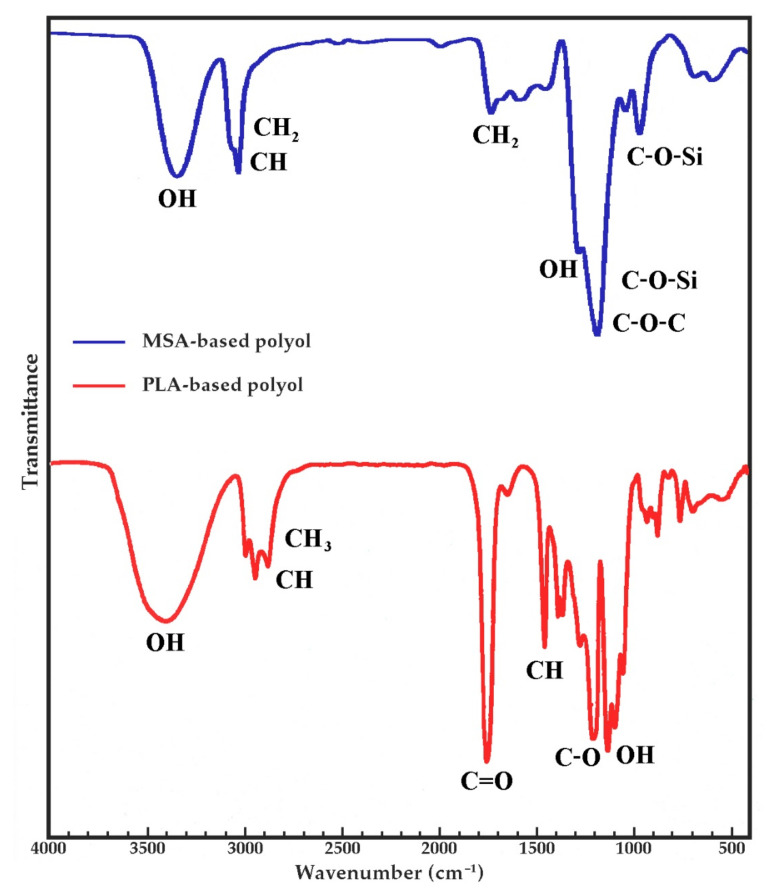
Fourier transform infrared (FTIR) spectra of MSA- and PLA-based polyols.

FTIR analysis confirmed the presence of characteristic groups in MSA- and PLA-based polyols. Figure 1 shows the spectra of both polyols.

In the FTIR spectrum of oligoetherol based on MSA (Figure 1) there was a wide band of valence vibrations of OH groups in the range of 3250–3600 cm^−1^, while the C-OH bands were observed at 1117 cm^−1^. There was also an intense band at 2868 cm^−1^ from the C-H stretching vibration. The high intensity C-O-C band at 1042 cm^−1^ coincided with the C-O-Si band. The presence of C-O-Si bonds confirmed the band at 934 cm^−1^ [29]. Analysis of spectrum of PLA-based polyol showed that there were characteristic bonds of the structure of lactide esters. The spectrum of this compound showed high band intensity at 3400 cm^−1^, which indicated the presence of O–H bonds in the hydroxyl groups. Bands at 2880–3000 cm^−1^ (stretching) and at 1360–1460 cm^−1^ (deformational) belonged to the C–H bond of the –CH_2_– and –CH_3_ group; at 1640–1760 cm^−1^ (stretching) belonged to the C=O bond of the ester group; at 1050–1270 cm^−1^ (stretching) belonged to the C-O bond of the ester group, and at 870–930 cm^−1^ belonged to the free carboxyl group [5].

**Figure 2:**
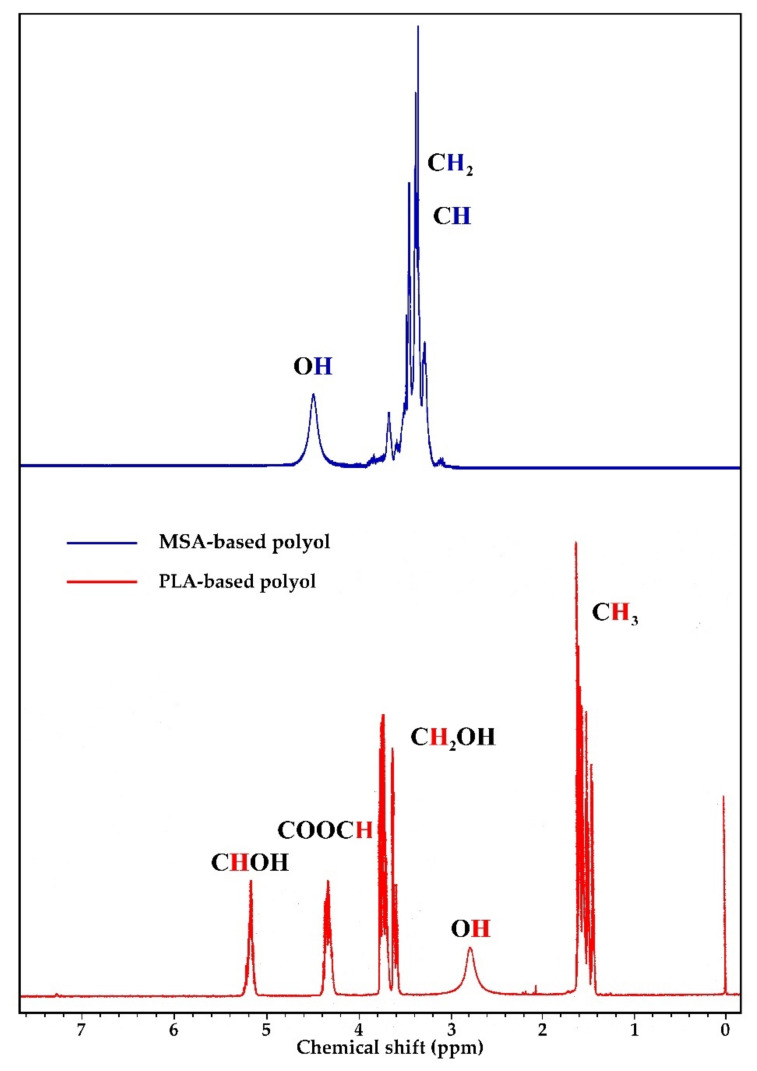
^1^H NMR spectra of MSA- and PLA-based polyols.

The ^1^H NMR spectra of the obtained polyol raw materials are shown in Figure 2.

In the spectrum of MSA-based polyol (Figure 2), the chemical shifts of protons from the methylene and methine groups formed by opening GL and EC rings were in the range of 3.2–3.6 ppm. Chemical shift of protons from the hydroxyl groups was at 4.5 ppm. Analysis of ^1^H NMR spectrum of PLA-based eco-polyol showed characteristic chemical shifts for: 1.43–1.44 ppm protons of methyl group from lactic acid monomers, 2.6–2.9 ppm protons of hydroxyl groups in macromolecules, 3.7–3.8 ppm protons of α-CH_2_ groups to the hydroxyl group (in glycol chain), 4.2–4.3 protons of α-CH groups to the ester group ppm, and 5.17 ppm protons of carbon in the α-position to the hydroxyl group (ending of polymer chain) [5,30,31]. The presence of protons of hydroxyl groups in the range of 2.6–2.9 ppm and at 4.5 ppm was demonstrated by adding D_2_O to the system and observing the disappearance of the mentioned signals [32]

### 2.2. Properties of Rigid PU Foam

#### 2.2.1. Foaming Process and Foam Morphology

Several preliminary foaming tests were carried out. During these tests, processing times (cream, free rise and tack free times) of the obtained foams were determined and their macroscopic characteristics were examined. The obtained results are presented in Table 2.

The optimal amount of eco-polyol in the mixture was 50 wt.% of the total amount of polyol raw materials in the foam formulation. The increase in the amount of this polyol in the formulation (over 50 wt.%) significantly deteriorated the properties of the obtained foams. This was mainly due to the increasing amount of linear PLA-derived structures in the polyurethane matrix. The introduction of this type of structure was associated with a decrease in cross-linking of the obtained material. Consequently, it also affected the deterioration of mechanical properties and a decrease in foam stiffness. During the preliminary tests, the influence of the amount of catalyst, surfactant, isocyanate raw material and blowing agent (CO_2_ from the reaction of water with an excess of isocyanate raw material) on the foaming process was also determined. Examining the influence of the amount of water on the course of the foaming process, it was noticed that the most advantageous amount of water in a polyurethane formulation was 2 wt.% of the total weight of polyol raw materials. The addition of higher amount of water (3 wt.% in formulation no. 1) resulted in obtaining a foam with irregular pore structure. This structure could significantly affect the performance properties of foam, e.g., increase its brittleness [33,34]. It was noted during preliminary tests that the amount of used isocyanate raw material had a significant influence on the properties of the obtained foams. In order to obtain a foam with good performance properties, the amount of isocyanate should be used so that the isocyanate index is 160. During investigating the optimal amount of catalyst and surfactant, it was found that, in an optimal formulation, their amount can be reduced in comparison with the reference formulation. The reduction of amount of these additives in the polyurethane formulation was mainly due to the high reactivity of the eco-polyol containing primary hydroxyl groups in its structure. Macroscopic tests of RPUFs showed that the best properties of rigid polyurethane foam (such as e.g., high stiffness, high regularity of pores or no defects on the material surface) had foam obtained on the basis of formulation no. 6. Selection of this foam as foam with optimal properties was also due to the lower consumption of additives (catalyst, surfactant). Therefore, foam no. 6 was used for further research.

The cream, free rise and tack free times for the optimal polyurethane foam were 40, 75 and 75 s, respectively. These times were longer than those for the reference foam, which were 30, 55 and 56 s, respectively. The elongation of processing times was a consequence of the reduction in the amount of catalyst in the formulation. This reduction was necessary because there were a large amount of primary hydroxyl groups in the reaction mixture. In the presence of the catalyst, the primary OH groups react much more rapidly with the isocyanate than the secondary or tertiary OH groups. This was indicated by the processing times of foams based on formulations no. 3, 4, 5 and 6. The elongation of all processing times was observed in them, with the reduction of the catalyst content in the formulation. This directly influenced the properties of the obtained material. The foams obtained on the basis of formulations no. 3 and 4 were characterized by high brittleness. This resulted in their cracking, e.g., during removal from the mold. In turn, the foams obtained on the basis of formulations no. 5 and 6 were more resistant to mechanical factors. However, the foam based on formulation no. 5 was obtained with higher amounts of catalyst and surfactant. It was important from an economic point of view, because higher consumption of raw materials increased the cost of production. Formation of the cellular structure of polyurethane foam is closely related to the foaming parameters and the qualitative composition of the formulation [9,35,36]. Sufficiently long reaction time allows for the simultaneous formation of an appropriate pore structure and prevents diffusion of the blowing agent from the inside of the obtained foam.

**Figure 3:**
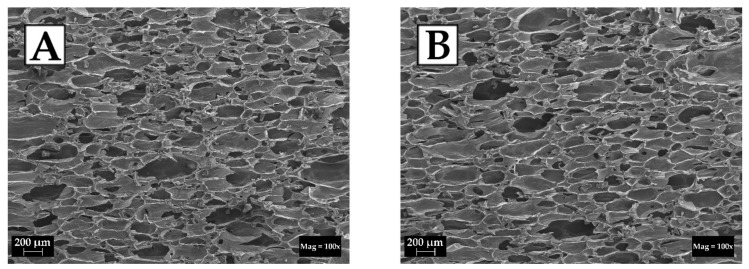
Micrographs of cellular structure of foams based on: (**A**) MSA-based polyol; (**B**) mixture of MSA- and PLA-based polyols.

The reference foam and the foam based on formulation no. 6 were tested by scanning electron microscopy (SEM). Statistical analysis (average pore size, average thickness of pore walls and average content of pores per area unit) was performed on the basis of the obtained micrographs. Selected SEM micrographs of the reference foam and foam no. 6 are shown in Figure 3, while the results of the statistical analysis are presented in Table 3.

It can be concluded—based on the SEM micrographs of the reference foam (Figure 3A) and the foam modified by eco-polyol based on PLA waste (Figure 3B)—that, in both cases, the pores had an oval shape. Both foams also had a large pore size distribution. This meant that the material contained pores with small diameters (>100 μm) and pores with diameters of approx. 200 μm. The large distribution of pore sizes affects the properties of the obtained materials [9]. The occurrence of an irregular pore structure influences, among others, on the value of the thermal conductivity coefficient. The higher share of pores with large diameters is, the easier they may also be subject to cracking. This leads to the release of the blowing agent from inside the pores and the introduction of atmospheric air in its place. This directly increases the thermal conductivity of foam. The thickness of the pore walls is also important. It is assumed that the thicker the pore wall is, the more resistant the pore is to cracking. A higher wall thickness may also have a beneficial effect on the mechanical properties of the foam [37,38]. In the case of the analyzed foams, the values of this parameter were at a similar level (14–15 μm). The wall thickness of the foam based on a polyol mixture was slightly higher. The content of pores per area unit was dependent on the pores size on it. Foam based on polyols mixture had a higher number of pores per 1 mm^2^ than the reference foam based on MSA-polyol. This was due to the larger distribution of pore sizes.

#### 2.2.2. Physico-Mechanical and Thermal Insulation Properties

Significant parameters determining the use of rigid polyurethane foams are their physico-mechanical properties. One of them is the apparent density of the foam. This parameter influences directly or indirectly on other material properties, e.g., compressive strength [9,39]. The results of the physico-mechanical tests are presented in Table 4.

The chemical structure and properties of polyol raw materials used for the synthesis of polyurethane foam have a significant impact on its properties [40]. Changes in all physico-mechanical properties were noted after using the mixture of MSA-based polyol and PLA-based eco-polyol. The use of polyol based on PLA waste in the polyurethane formulation decreased the apparent density from 95.5 kg/m^3^ for the reference foam to 89.3 kg/m^3^ for the foam based on mixture of polyols. The use of glycolysis reaction to recycling of PLA waste resulted in obtaining eco-polyol raw material, which contained long linear chains. The addition of such component in a mixture with a polyol with a branched chemical structure, to the foam matrix resulted in a reduction of its apparent density. The decrease in this parameter was due to the decrease in the cross-linking degree of the polyurethane. The use of materials with lower apparent density is more advantageous from an economic point of view, because it reduces the price of the final product. Czupryński et al. used the glycolysis reaction to convert waste from RPUFs into a polyol intended for the synthesis of rigid polyurethane/polyisocyanurate foams. They noticed the opposite dependence than in the case of using an eco-polyol based on PLA glycolysis. A small addition of PU foam glycolysate increased the apparent density of foams from 37 kg/m^3^ to 66 kg/m^3^. The reason for this was the obtaining of a polyol raw material with a branched chemical structure. Moreover, the glycolysate molecules contained nitrogen heteroatoms, which significantly influenced the process of polyurethane material synthesis, and, thus, its structure [41].

The compressive strength of rigid polyurethane foam is a very important parameter when it is used as a thermal insulator. During exploitation, this material is constantly subjected to compressive loads which it must withstand. In addition, the compressive strength also affects the stability of linear dimensions and geometric volume of foams [5]. Deformation of the foam can often be observed during use, especially when it is exposed to high and low temperatures. This is due to changes in the pressure of the blowing agent enclosed in the foam pores (in this case, carbon dioxide). These pressure changes are mainly caused by changes in temperature around the foam. It is assumed that the polyurethane foam will not deform if its compressive strength is higher than 100 kPa. This value represents the maximum pressure difference that can occur between the atmospheric pressure and the pressure inside the foam pores, assuming complete condensation of the blowing agent [4,42]. A slight increase in compressive strength was noted during the research, from 486 kPa for the reference foam to 493 kPa for the foam obtained from formulation no. 6. The change of this parameter was slight and it can be assumed that it was within the measurement error range. Thus, it can be concluded that the modification of a formulation containing MSA-based polyol with a PLA-based eco-polyol did not affect the compressive strength value. In general, polyol raw materials obtained in the glycolysis process significantly reduce the mechanical strength of RPUFs based on them [5,41,43,44]. This is largely due to the heterogeneity of the chemical composition of the obtained polyols, which directly affects the process of polyurethane synthesis and its properties.

The use of a PLA-based polyol in the formulation changed the water absorption values. It was noted that the modification of the formulation with eco-polyol led to reduction of water absorption from 3.82, 4.07 and 6.68% for the reference foam to 0.59, 2.74 and 5.14% for the modified foam after 5 min, 3 h and 24 h of immersion, respectively. Low water absorption of RPUFs is a favorable parameter because it allows for using this foam as an insulating material in places with high humidity, without deteriorating the insulating properties [45]. The inverse dependence concerning water absorption was observed in the case of the use of glycolysis products of PU foam waste. In this case, the addition of recycled polyol to the polyurethane formulation resulted in an increase in water absorption. The reason for the increase in this parameter was the complex structure of the polyol molecule obtained from PU recycling. This promoted the formation of open-cell structure of foams [45,46]. Increasing water absorption is an undesirable phenomenon because it reduces the thermal insulation properties of PU foams. The lower water absorption could indicate that the foam modified by eco-polyol was characterized by a higher content of closed cells in comparison with the reference foam. This was also confirmed by the analysis of the SEM micrographs (Figure 3).

An important parameter of RPUFs intended for thermal insulation application is the value of the thermal conductivity coefficient (λ). The value of this parameter depends mainly on the type of raw materials used in the formulation and the cell structure of the finished product. The thermal conductivity coefficient of the reference foam based on MSA-polyol was equal 36.8 mW/(m·K). The use of a mixture of polyols based on MSA and PLA for the synthesis of RPUF reduced the value of λ to 34.4 mW/(m·K). The reason for this was the increase in the content of closed cells in the modified foam from 78% for Ref. foam to 83% for foam no. 6. This was also confirmed by the SEM micrographs analysis. The obtained values of λ for the reference and modified foam were within the industrial standards for this type of material (30–40 mW/(m·K)) [9]. Usually, the addition of recycled polyol (e.g., from the glycolysis of polyurethane foam) into the formulation leads to obtain PU foams with a higher thermal conductivity coefficient (35–40 mW/(m·K)) [41,43]. The value of the thermal conductivity coefficient is also strongly dependent on the type of used blowing agent. Carbon dioxide generated in situ in the reaction of excess isocyanate raw material with water was used as a blowing agent in the research. The value of the thermal conductivity coefficient of this blowing agent was 16.4 mW/(m·K), which had a significant impact on the λ of the obtained foams. Using a physical blowing agent, the value of which is about 10 mW/(m·K), would reduce the λ coefficient of foams to the range of 20–30 mW/(m·K) [4,9,47].

#### 2.2.3. Thermal Resistance Properties

**Figure 4:**
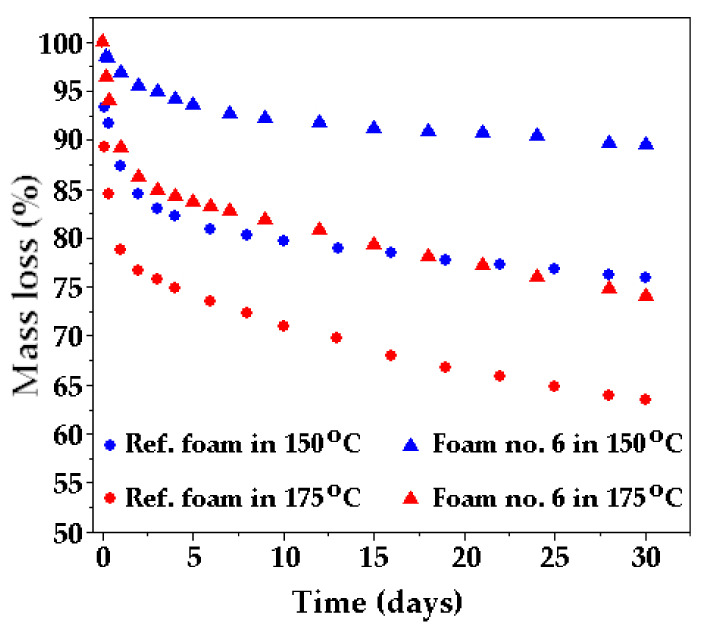
Comparison of mass loss of foams during 30-day exposure at elevated temperature.

Thermal stability determines thermal durability and heat resistance. Polymers with higher thermal stability are characterized by: higher melting, softening and thermal decomposition points, lower mass loss during heating and a higher heat deflection temperature under load, without deteriorating their basic properties determining their functionality. The thermal stability of RPUFs depends mainly on the polyurethane matrix, which is formed as a result of the reaction of the polyol raw material with the isocyanate [48]. Reference foam based on MSA-based polyol and foam no. 6 based on a polyol mixture were tested for static thermal resistance at 150 and 175 °C. Mass loss was measured during the test. The dependence of mass loss of foams and thermostating time is shown in Figure 4. A gradual decrease in the mass of foam was observed during the thermostating. The highest mass losses were observed during the first day of heating. 

The advantage of adding PLA-based polyol to the polyurethane formulation was an increasing the thermal resistance of foam. Mass losses of the foam based on the mixture of MSA- and PLA-based polyols were always lower than mass losses of the reference foam at the measurement temperatures (Figure 4). The mass losses after 30-day exposure at the temperatures of 150 and 175 °C were 2.5 times and 1.4 times lower, respectively, than those for the foam unmodified by PLA-based polyol. The reason for this could be the cross-linking of polyurethane (at a temperature of 150 °C) or the destruction of polyurethane (at a temperature of 175 °C), which was related to the mass loss and the formation of carbon layer on the surface of foam.

**Figure 5:**
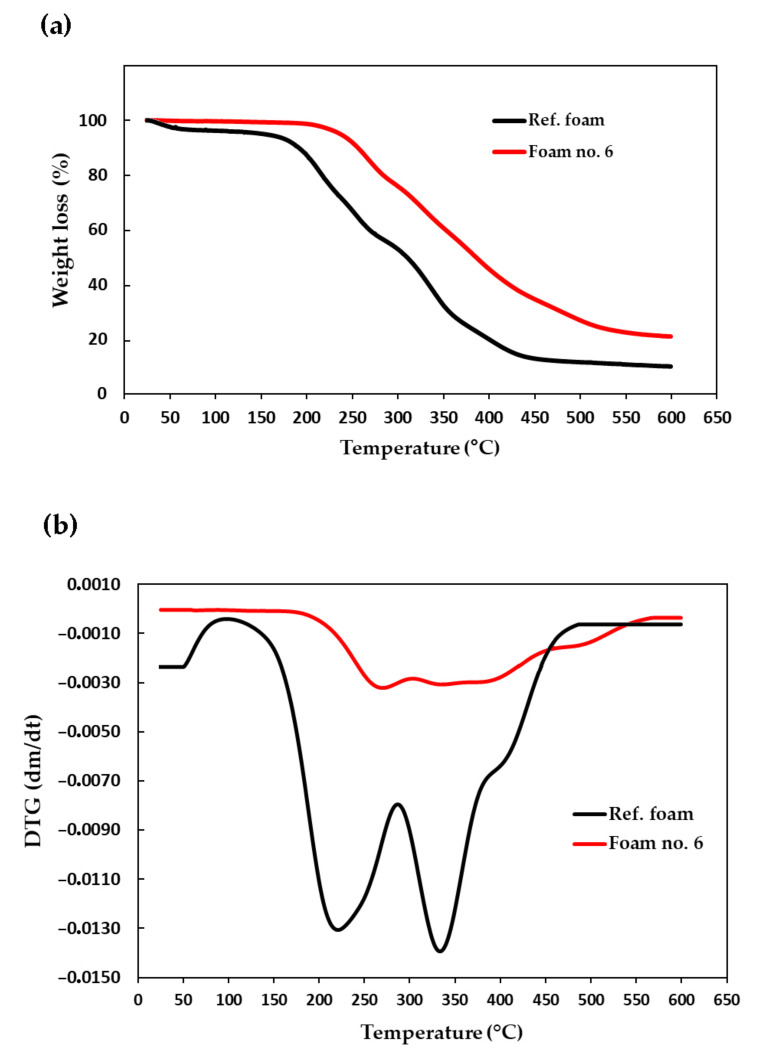
TG (**a**) and DTG (**b**) curves of reference foam (Ref.) and foam based on mixture of polyols (no. 6).

The differential scanning calorimetry (DSC) analysis showed that the glass transition temperature (T_g_) of the foam no. 6 was 111 °C (Table 5). This meant that it was significantly reduced in comparison with the glass transition temperature of the reference foam, which exceeded 200 °C. The reason for this was the presence of long oligomeric chains of eco-polyol, which made the foam more flexible. At the same time, the introduction of long and linear polyester chains into the polyurethane matrix did not reduce the resistance to increased temperatures. Chuang et al. [49] observed a similar dependence regarding the influence of polyol chain length on thermal stability. The flexible structure of such chains protected, for example, against deformation caused by migration of the blowing agent at elevated temperature [50]. Thermal analysis carried out with the dynamic method showed that foam no. 6 started to decompose at 230 °C (5% weight loss, Table 5), which confirmed its higher thermal resistance in comparison with the reference foam obtained only from MSA-based polyol (5% mass loss took place at 167 °C). Detailed thermograms of the reference foam and foam no. 6 are shown in Figure 5. 

**Figure 6:**
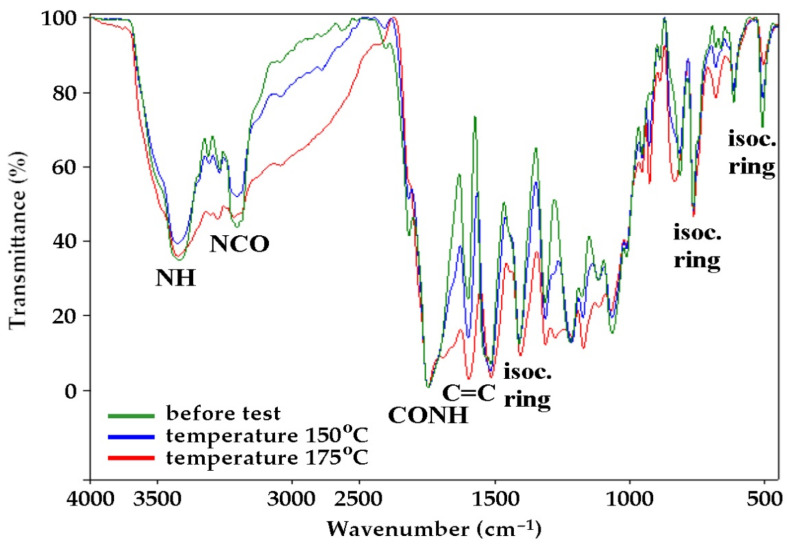
FTIR spectra of foam obtained from a mixture of polyols based on PLA and MSA before and after thermostating at the temperature of 150 °C and 175 °C.

Rapid decomposition of the foams was observed in range from 220 to 270 °C (Figure 5b). It was associated with thermal degradation of the oxyalkylene chains in MSA-based polyol and urethane bonds. It also concerned the degradation of ester bonds in the eco-polyol structure (bonds between lactic acid monomers and bonds between of lactic acid monomer and MEG molecule) [51]. Further decomposition in the temperature range from 340 to 390 °C was associated with the degradation of isocyanurate rings obtaining from the trimerization of the isocyanate raw material. The value of isocyanate index (>100) was the confirmation of the formation of isocyanurate rings during the foam synthesis (PU/PIR foam was obtained). This value indicated that the amount of reacting hydroxyl and isocyanate groups was not stoichiometric. This meant that, in the reaction system, there was an excess of isocyanate, which was used partly in the reaction with water and partly in the trimerization reaction. The presence of isocyanurate rings was also confirmed by the analysis of FTIR spectra of foams (Figure 6). It showed bands indicating the presence of isocyanurate rings at 1410, 780 and 525 cm^−1^ (from the vibration of the entire isocyanurate ring), while there were not any bands of unreacted isocyanate groups at 2275 cm^−1^.

The modification of the formulation used in the research consisted in adding long flexible chains derived from PLA-polyol into a rigid polyurethane foam containing silicon heteroatoms derived from MSA-polyol. The foam based on the modified formulation decomposed at elevated temperatures, and the reactions in the condensed and gas phases contributed to the formation of a char layer on the surface of obtained material. The formation of a carbonized layer and a layer of silicate glass limited the mass loss and increased the thermal stability of polyurethane [52].

#### 2.2.4. Flammability of Rigid PU Foam

The combustion process of the polymer material is accompanied by the reactions taking place in the flame and the thermal decomposition of the matrix. The intensity of these processes depends largely on the reactions taking place on the surface of the solid phase. Large amounts of flammable gases, which support combustion process, are produced as a result of endothermic degradation reactions of polymer. The progressing degradation is supported by the heat of strongly exothermic oxidative reactions taking place in the surface layer between the solid and gas phases. Then, heat penetrates inside the polymer, which contributes to increasing the temperature of the material. The increase in temperature enables the initiation of further degradation processes, e.g., pyrolysis, which results in polymer gasification [53,54].

In the case of polyurethane foams, there is a varied and complex chemical composition that can lead to a wide variety of chemical reactions during the combustion process. The combustion of polyurethane material is also promoted by a large number of carbon-hydrogen bonds, a porous structure and low density. The use of flame retardant compounds allows to significantly change the fire performance of polyurethane materials, making them safer during exploitation [55]. A modern approach to the problem of flammability of polyurethane plastics consists in cessation of the use of flame retardants based on halogen compounds (bromine, chlorine) or aluminum and magnesium hydroxides for those whose chemical composition will allow to create a barrier that cuts off the oxygen supply from the fire source during a fire [56,57]. Such a mechanism is demonstrated by silicon compounds that are able to create a silicate glass barrier on the surface of the burning polymer. The purpose of using a polyol composition consisting of PLA-polyol and MSA-polyol in the formulation was to produce silicate glass on the surface of foams subjected to flammability tests. Polyurethane foams are very often used as thermal insulation materials (e.g., for transmission pipelines), due to the excellent insulating properties, resistance to chemical and biological factors and ease of use. The effectiveness of the properties of materials is limited by the its maximum application temperature at which thermal insulation can still perform its functions without creating a fire hazard. Knowledge about the fire resistance of the used material is the basis for planning its application. Therefore, during this research, the flammability of polyurethane foams after 30-day thermostating at 150 and 175 °C and not being subjected to temperature exposure was assessed. The determined fire parameters are presented in Table 6.

It can be concluded, based on results of horizontal test, that the burning rate of the foam based on polyol mixture was 1.37 mm/s. This meant that it burned slower than the reference foam obtained from MSA-based polyol (2.1 mm/s). The foam based on a mixture of polyols also left about 50% of char layer after burning, unlike the reference foam, which melted and dripped when burned. The foams subjected to exposure at a temperature of 150 °C burned only in the flame and immediately went out after its removal. On the other hand, the foams thermostated at 175 °C did not ignite at all by the flame. The LOI of the foam obtained on the basis of PLA-polyol and MSA-polyol was 20.9% and it was slightly lower than LOI of the reference foam (21.1%). The LOI values of the foams increased after the high temperature exposure. The research showed that the foams subjected to long-term heating had a reduced flammability, which was the advantage resulting from the change in its structure. It was the effect of adding long and linear polyester chains into the polyurethane matrix, which were relatively resistant to high temperatures. Slow carbonization took place at elevated temperature, which caused difficulties in combustion process [58]. The formation of char layer by limiting the access of the flame to the deeper layers of material prevented the generation of low-molecular organic compounds that support the burning process. The char layer effectively hindered the diffusion of oxygen and the transfer of mass and heat between the flame and the polyurethane matrix. Thus, the self-supporting combustion of the polyurethane foam was stopped. This was indicated by an increase in the band intensity at 1610 cm^−1^ in the FTIR spectra of the modified foam along with increasing the temperature of its thermostating (Figure 6). The obtained results suggested that this foam could be used as thermal insulation up to the temperature of 175 °C. Moreover, heating the foam in the air atmosphere was accompanied by oxidation processes. Such possibility was indicated by an increase in the intensity of the bands at 3400 cm^−1^ and 1150 cm^−1^, derived from the O-H and C-OH valence vibrations in the FTIR spectra of foam based on the MSA- and PLA-polyol. An additional advantage of the obtained foam is that the foam subjected to temperature exposure not only reduced its flammability but also increased the compressive strength in relation to non-thermostated foam. It is worth noting that it happened with a simultaneous slight mass loss. The compressive strength of the PLA-polyol-modified foam after thermostating at 150 and 175 °C increased by 163 and 430%, respectively, which was its advantageous utility feature. The reason for this was the presence of silicon atoms in the foam structure, because similar dependence was observed to a slightly greater extent in the reference foam. 

Analysis of the combustion test performed in the cone calorimeter provided significant information about flammability of foams. Time to ignition (TTI) is one of the most important parameter of material thermal resistance. The higher this parameter is, the longer it takes for the material to heat, ignite and initiate a fire [59]. Due to the low thermal conductivity of polyurethane foams used in thermal insulation, these materials react quickly to the applied heat flux [60]. Therefore, the surface of the test specimens was rapidly heated after being exposed to the external heat flux of the cone calorimeter. This led to the formation of pyrolysis products in a very short time and, thus, to the rapid development of the combustion process. Studies have shown that the addition of a mixture of polyols (based on MSA and PLA) into the foam composition was advantageous because the modified foam ignited much more difficult than the reference foam. Its TTI was 26 s, while the TTI of reference foam was only 7 s (Table 7). Additionally, the total time to flameout (TTF) of the foam based on MSA- and PLA-polyol was more than twice as long (159 s) than TTF of the reference foam (75 s).

**Figure 7:**
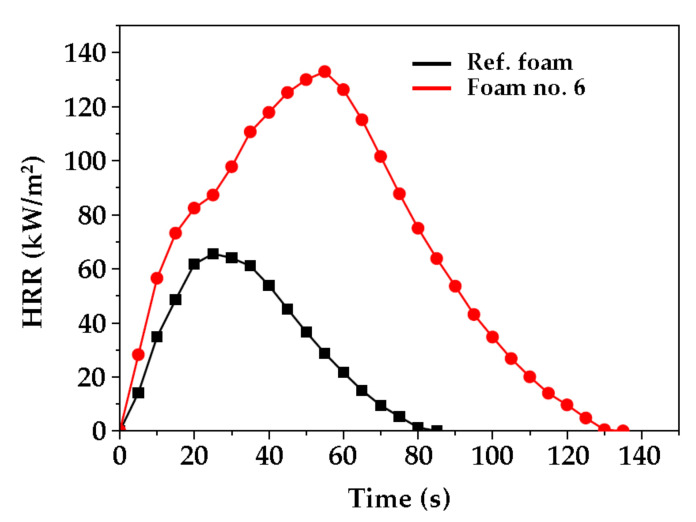
Comparison of the heat release rate (HRR) of the reference foam (Ref.) and foam based on MSA- and PLA-polyol (no. 6) as function of time.

**Figure 8:**
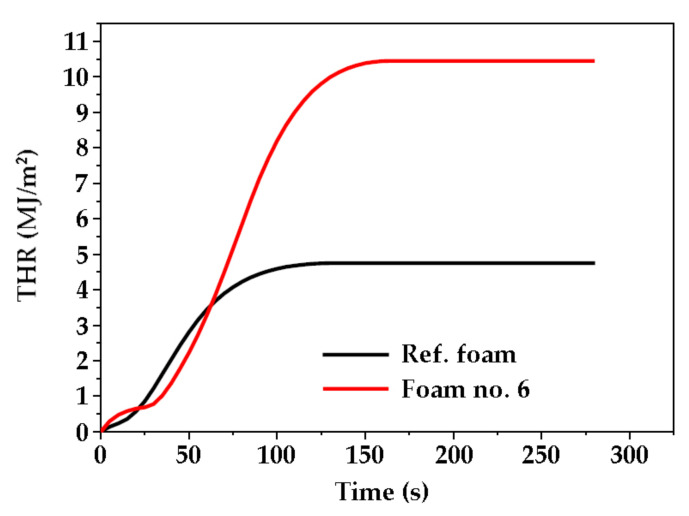
Total heat released (THR) of the reference foam (Ref.) and foam based on MSA- and PLA-polyol (no. 6) as function of time.

Curves characterizing the course of heat release rate (HRR) for the reference foam and modified foam are shown in Figure 7, while the total heat release (THR) for both foams is shown in Figure 8.

The foam modified by a mixture of polyols was characterized by a higher heat release rate. The maximum HRR was achieved after a longer time, and the amount of heat released was approximately twice as high than the reference foam. This was due to the higher share of carbon and hydrogen atoms in foam matrix caused by the presence of eco-polyol structures. Despite the large amount of heat released, the foam modified by mixture of polyols burned slowly (Figure 7). It was found that the percentage mass loss of the foam no. 6 was about 10% higher than that of the reference foam obtained from MSA-based polyol. The effective heat of combustion (EHC) depended on the type of burned foam (Table 7). The foam modified by the polyol mixture gave off less heat (44.5 MJ/kg) than the reference foam (53.7 MJ/kg). This could be related to the lower apparent density of foam no. 6. In the case of the total heat release (THR), it was noticed that the foam modified by eco-polyol emitted more heat per area unit (m^2^) during combustion than the reference foam (Figure 8).

The values of fire parameters of reference foam and foam no. 6 confirmed that the reason for the obtained fire characteristics was the structure of the polyurethane foams and their chemical composition. The addition of reactive silicon compounds and the reduction of matrix cross-linking due to the increase in the number of linear chains significantly improved the thermal stability and fire resistance of polyurethane foams. This was because the silicon compounds reduced the amount of flammable organic components in the foam, and silica formed in the combustion process was a layer on the polyurethane surface that inhibited the heat flow. A similar dependence between the flammability of polyurethane materials and the content of silicon compounds was noticed by Przekop and Marciniec, Semenzato et al. and Lubczak and Chmiel [61,62,63].

### 2.3. Results of Susceptibility to Biodegradation Test

**Figure 9:**
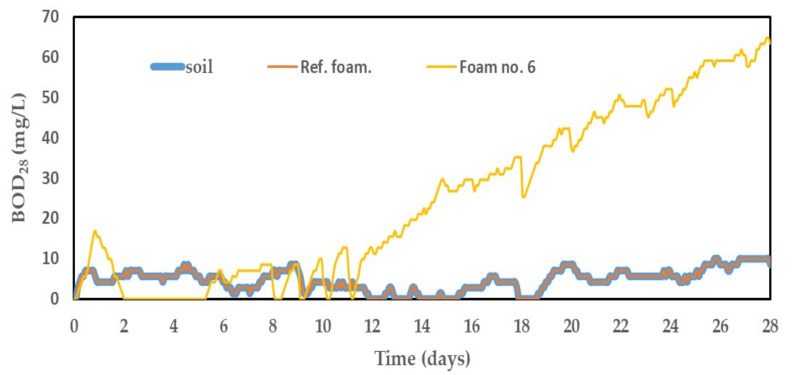
Results of biochemical oxygen demand of foams during measurement.

A big problem of the modern world is the large amount of generated waste. This applies to all areas of everyday life as well as industrial production. Plastic waste is the biggest problem. The increase in the consumption of polymeric materials contributed to the increase in the amount of generated waste from these materials [5,9]. This problem is particularly evident with polyurethanes. These materials are relatively resistant to degradation due to various modifications. Therefore, their waste is very permanent in the environment and its natural decomposition can take hundreds or even thousands of years [9]. Therefore, it is necessary to look for such solutions, which allow the decomposition of this waste in the shortest possible time. A very interesting raw material from this point of view is PLA. It belongs to the group of biodegradable polymers due to its natural origin. It is assumed that the products obtained on the basis of PLA (and, at the same time, the waste from this material) will also be biodegradable under certain conditions [6]. A study of susceptibility to biodegradation of the reference foam and the foam based on a mixture of polyols in soil environment was performed in order to check the effect of eco-polyol on biodegradation. Powdered foams were introduced into the system containing the previously prepared soil solution. Changes in BOD of the analyzed samples were monitored for 28 days. The course of BOD changes during the measurement is shown in Figure 9.

The final BOD values were read for both samples after 28 days. The mass shares of carbon, hydrogen, oxygen, silicon and nitrogen was calculated based on the elemental analysis. The obtained values are presented in Table 8. Theoretical oxygen demands for both foams were calculated on their basis. The degree of biodegradation (D_t_) of the tested foams was calculated based on measured BOD after 28 days and the calculated TOD. The obtained results are presented in Table 9.

The susceptibility to biodegradation test of the reference foam based on MSA-polyol showed that it did not undergo any processes in the soil environment. This was evidenced by the BOD_28_ value, which was 0 mg/L. This meant that the foam was neutral to the chemical and microbiological factors contained in the soil. The reason for this may also be the presence of carbon of petrochemical origin. The literature reports that petrochemical carbon is much more resistant to biodegradation than carbon of natural origin [64]. All ingredients used in the synthesis of reference foam were of petrochemical origin. Important also is the fact that the reference foam (unlike the foam no. 6) did not contain ester groups in its structure. These groups are much easier to biodegrade. Thus, it made the reference foam not biodegradable (D_t_ = 0%). It was different in the case of the foam obtained on the basis of a mixture of polyols. Figure 9 shows a clear increase in BOD over time. It reached a value of 54.90 mg/L after 28 days. This had a direct influence on the degree of biodegradation of this foam, which, finally, was 68.41%. This was due to the presence of biodegradable PLA chains in foam matrix. The process of PLA glycolysis contributed to the shortening of the polymer chains, which favored even faster biodegradation of this compound. Such a high degree of biodegradation meant that the hard-to-degradable polyurethane structures were broken into smaller structures in the place where the PLA-based polyol was incorporated. The fragmentation of the matrix contributed to a decrease in the degree of polyurethane cross-linking, which could cause the degradation of fragments that were previously difficult to degrade. Of course, it should be remembered that this study was a simplified analysis of the susceptibility to biodegradation. This analysis only referred to environmental parameters and did not take into account the microbiological aspects of the testing environment. The scale of the measurement was also important, because the ratio of tested material to the environment was 1:1500. However, it was completely sufficient at this stage of the research, because it provided valuable information that PLA-based eco-polyol may affect the biodegradation of PLA-based materials.

## 3. Materials and Methods 

### 3.1. Materials

A 40% solution of water glass (sodium silicate, Dragon, Skawina, Poland) and a 35% solution of hydrochloric acid (POCh, Gliwice, Poland) were used to obtain MSA. In a further step, it was used for the reaction with glycidol (GL, Sigma-Aldrich, Saint Louis, MO, USA) and ethylene carbonate (EC, Sigma-Aldrich, Saint Louis, MO, USA) in the presence of potassium carbonate (POCh, Gliwice, Poland) as a catalyst. Eco-polyol was obtained as a result of the glycolysis reaction of PLA waste from various sources with ethylene glycol (MEG, Chempur, Piekary Śląskie, Poland) in the presence of a catalyst—zinc stearate (ZC, Chempur, Piekary Śląskie, Poland). A mixture of two newly synthesized polyols was used for the synthesis of rigid polyurethane foams. Besides new polyol raw materials, polymeric diphenylmethane diisocyanate (pMDI, Merck, Darmstadt, Germany) was used as a isocyanate raw material for synthesis of RPUFs. The other additives for the preparation of RPUFs were triethylamine (TEA, Sigma-Aldrich, Saint Louis, MO, USA) as a catalyst, Silicon L-6900 (Momentive, Strongsville, OH, USA) as a surfactant, and distilled water to form a blowing agent.

### 3.2. Synthesis of Polyol Based on Metasilicic Acid

The first step of the synthesis of MSA-based polyol was the preparation of metasilicic acid. A total of 100 g of a 40% water glass solution was added into a 200 mL beaker for this purpose. Then, 65 g of concentrated hydrochloric acid was added in portions, with constant stirring, until the pH of reaction mixture was 6.0. A white colloidal precipitate was formed in the solution as a result of the double replacement reaction. The obtained precipitate was separated from the solution and washed with distilled water until the pH was neutral. After that, it was dried at room temperature and then in a vacuum oven at 80 °C until constant weight was obtained.

**Figure 10:**
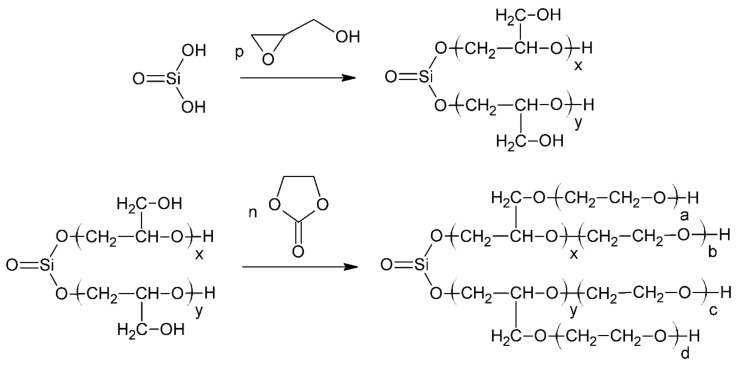
Scheme of synthesis of polyol based on metasilicic acid (where: p = x + y and n = xa + b + c + yd).

In the next step, 7.8 g (0.1 mol) of synthesized MSA and 29.6 g (0.4 mol) of GL was added into a 250 cm^3^ three-necked flask equipped with a reflux condenser, a mechanical stirrer and a thermometer. The reaction system was heated to a temperature of 120 °C. The exothermic effect of the conducted reaction caused the additional heating of the reaction mixture to 170 °C. The reaction was carried out for 1.5 h at 180 °C. The progress of reaction between MSA and GL was monitored by measuring the content of epoxide groups in accordance with ISO 3001:1999. The reaction mixture was cooled to room temperature after end of the reaction and 26.3 g (0.3 mol) of EC and 0.35 g (0.0025 mol) of potassium carbonate catalyst were added. The system was reheated to 180 °C and reacted at this temperature for 14 h. Slight gas evolution from the reaction mixture was observed during the synthesis. The progress of reaction between semi-product and EC was monitored using barium hydroxide method in accordance with the method described in the article [65]. The end of the reaction was determined from the determination of the unreacted EC content. A MSA-based polyol was obtained in which the molar ratio of the reactants was 1:4:2.6 for MSA:GL:EC, respectively. The scheme of the MSA-based polyol synthesis reaction is shown in Figure 10.

### 3.3. Synthesis of Eco-Polyol Based on PLA Waste

**Figure 11:**
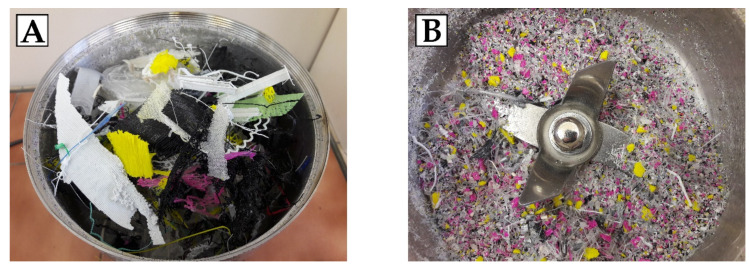
Appearance of PLA waste used for the synthesis of eco-polyol: (**A**) before grinding; (**B**) after grinding.

PLA waste before the reaction was ground in a laboratory mill to grains with a diameter less than 5 mm. This operation was necessary to be able to add the polymeric raw material to the reaction vessel. The appearance of the waste used in the synthesis of eco-polyol is shown in Figure 11.

**Figure 12:**
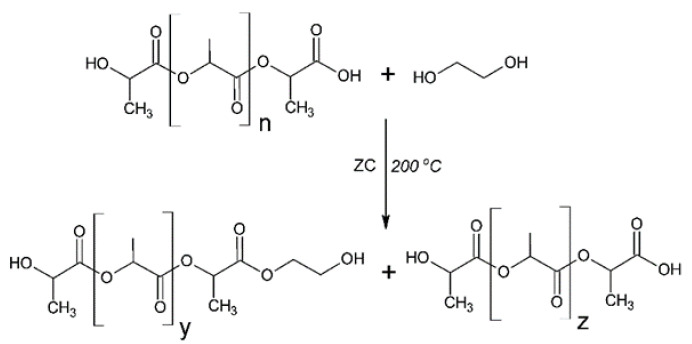
Scheme of synthesis of eco-polyol based on PLA waste (n = y + z).

A total of 50.0 g of ground PLA, 50.0 g of MEG and 0.01 g of ZC were put into the three-necked flask with a reflux condenser, a thermometer and a mechanical stirrer. The reaction mixture was heated to 200 °C with continuous mixing of the stirrer (700 rpm). PLA was liquefied at this temperature. The glycolysis reaction (Figure 12) was carried out for 3 h, and then, the system was cooled. The new polyol compound was filtered after cooling and prepared for testing [66].

### 3.4. Synthesis of Rigid Polyurethane Foams

Preliminary studies of a mixture of polyols based on MSA and PLA have shown that the favorable performance properties of the RPUFs were obtained when the mass ratio of MSA-polyol:PLA-polyol was equal 1:1. A series of test foams were obtained in order to determine the optimal formulation of RPUFs based on the mixture of these polyols. The foam with the most advantageous processing parameters and macroscopic properties (e.g., high stiffness, no structural defects, regularity of pores) was selected for further research. Foam based only on MSA-polyol was used as the reference foam. Rigid polyurethane foams were obtained using the one-step method from two components system (A and B). Component A was a mixture of polyols with additives (water, catalyst and surfactant). Component B was pMDI. The amount of polyol raw materials and the isocyanate was selected taking into account the isocyanate index in the reaction mixture. For the test formulations it was 160 and 170, respectively. The contents of the additives were calculated per 100 g of polyols. Component B was added to component A, and then, the entire mixture was intensively mixed with a mechanical stirrer (1000 rpm) for 10 s. After that, the reaction mixture was poured into an open mold, where was a free rise of the foam in the vertical direction. The foams were thermostated after removing from the mold for 4 h at 120 °C. The formulations of the obtained polyurethane foams are presented in Table 10.

### 3.5. Tests Methods

#### 3.5.1. Properties of Polyols

Physicochemical, analytical and spectroscopic tests were performed on the MSA- and PLA-based polyols. This was for determining their suitability for the synthesis of polyurethane materials.

##### Analytical Tests of Polyols

The acid number (AN) of polyols was determined by titration with 0.1 M NaOH solution in the presence of phenolphthalein in accordance with PN-EN ISO 660:2010. The hydroxyl number (HN) of polyols was determined by acylation with acetic anhydride in xylene in accordance with PN-93/C-89052.03. An excess of acetic anhydride was titrated with 1.5 M NaOH solution in the presence of phenolphthalein. Elemental analysis of C, H, N and O were made with Carlo Erba EA1108 elemental analyzer (Thermo Fisher Scientific Inc., Waltham, MA, USA). Amount of silicon was determined spectrophotometrically in the form of SiO_2_ after previous mineralization of MSA-based polyol. Molecular weight (number-averaged and weight-averaged) and polydispersity of polyols were determined by gel permeation chromatography (GPC) using the following parameters: 25 ± 0.1 °C temperature, 1 cm^3^/min volume flow of eluent, 20 mL, volume of inlet chamber, 4–5 mg/cm^3^ polymer concentration, 30 min analysis time, eluent: *N*,*N*-dimethylformamide, calibration reference: polystyrene (a detailed description of GPC of polyols is contained in Supplementary Materials).

##### Physicochemical Properties of Polyols

The density of polyols was measured at 25 °C (298 K) in an adiabatic pycnometer in accordance with ISO 758:1976. The viscosity of the new compounds was determined by using a digital rheometer (Fungilab Inc., New York, NY, USA) at 20 °C (293 K). The measurements were carried out by using a standard spindle (DIN-87) working with the bushing (ULA-DIN-87). Maintaining a constant temperature of measurement was provided by a thermostat connected to the water jacket of the sleeve. The pH value was measured using a microprocessor laboratory pH-meter (ORP/ISO/°C) with RS22C connector (Hanna Instruments, Woonsocket, RI, USA). The color and smell of obtained polyol compounds were tested organoleptically.

##### Spectroscopic Tests 

New polyols were tested in FTIR spectroscopy using Nicolet iS20 (Thermo Scientific, Waltham, MA, USA) spectrophotometer in range from 400 to 4000 cm^−1^ and in ^1^H NMR spectroscopy using a Brücker NMR Ascend III spectrometer with a frequency of 400 MHz in D_2_O, as a solvent.

#### 3.5.2. Properties of Rigid Polyurethane Foams

##### Foaming Process

The foaming process was analyzed in accordance with ASTM D7487 13e^1^—Standard Practice for Polyurethane Raw Materials: Polyurethane Foam Cup Test. Cream, free rise and tack free times were measured during obtaining RPUFs by an electronic stopwatch.

##### Morphology of RPUFs

Microstructure of pores structure was analyzed by scanning electron microscope (SEM) HITACHI SU8010 (Hitachi High-Technologies Co., Tokyo, Japan). The studies were performed at the accelerating voltage in range from 10 to 30 kV, with the working distance of 10 mm and magnification of 100×. Statistical analysis of pore sizes, wall thickness and content of pores per area unit were carried out on the basis of obtained micrographs using ImageJ software.

##### Physico-Mechanical Properties

The apparent density of the foams was determined in accordance with ISO 845:2006. The ratio of the foam weight to the geometrical volume of the cube-shaped samples with a side length of 50 mm was calculated. Compressive strength was determined using the universal testing machine Instron 5544 (Instron, Norwood, MA, USA) in accordance with ISO 844:2014. The highest load causing a 10% decrease in the height of the foam in relation to the initial height (in foam rise direction) was determined. The volumetric water absorption was determined in accordance with ISO 2896:2001 after 5 min, 3 h and 24 h.

##### Thermal Resistance

Thermal resistance of modified foams was determined both by static and dynamic methods. In static method the foams were heated at 150 and 175 °C with continuous measurement of mass loss. Thermal analysis of foams in a dynamic method were performed using TGA/DSC thermal analyzer Mettler Toledo 822e (Mettler-Toledo, Columbus, OH, USA) with STARe software (Mettler-Toledo, Columbus, OH, USA). Measurement was performed in ceramic crucible in temperature range of 20–600 °C with heating rate of 10 °C/min, under air atmosphere. Sample weight was about 100 mg. The glass transition temperature (T_g_) of the foams was also determined using this device.

##### Thermal Conductivity Coefficient

Thermal conductivity coefficient (λ) was measured with ISOMET 2114 apparatus (Applied Precision Ltd., Rača, Slovakia) by inserting the cylindrical probe with a diameter of 4 cm and a length of 8 cm into the foam.

The content of closed cells was determined in accordance with ISO 4590:2016-11 using the helium pycnometer AccuPyc 1340 with the FoamPyc software. This software calculated the content of closed cells based on the measurement of pressure changes in the test chamber.

##### Flammability of RPUFs

Flammability tests of the obtained foams were performed using three methods: horizontal burning test, limiting oxygen index (LOI) and mass loss cone calorimeter. A horizontal burning test was carried out in accordance with PN-EN ISO 3582:2002/A1:2008. Foam samples with dimensions of 150 × 50 × 13 mm were weighed and placed on a horizontal standard support. Then they were marked with a line at a distance of 25 mm from the edge of the sample. The foam sample was set on fire (from the opposite edge than marked) by a Bunsen burner with blue flame of 38 mm high for 60 s. After this time, the burner was turned off and the time of foam burning was measured by electronic stopwatch. Measurement of the free burning time of the foam was carried out until the marked line was reached or the flame was gone. After that, the samples were weighed again. The burning rate was calculated according to the Equation (1) if the sample was burned totally:

(1) $$v=\frac{125}{t_{b}}$$

If the sample stopped burning, Equation (2) was used:

(2) $$v=\frac{L_{e}}{t_{e}}$$

where *L_e_*—length of the burned fragment measured as the difference between the sample length and the length of the unburned fragment (mm); *t_b_*—flame spread time measured from the edge of the sample to the end mark (s); *t_e_*—time of flame cessation (s). If the burned fragment is 125 mm long, the foam is considered flammable. The mass loss (Δ*m*) after burning was calculated from the Equation (3):

(3) $$\Delta m=\frac{m_{0}-m}{m_{0}}\times 100\%$$

where *m*_0_ and *m*—the sample masses before and after burning, respectively.

The limiting oxygen index flammability tests were performed using Concept Equipment LOI apparatus (Concept Equipment Ltd., Arundel, UK) in accordance with ASTM D 2863-1970. The LOI value is the limit concentration of oxygen in a mixture of oxygen and nitrogen that is sufficient to sustain the combustion of the sample. The LOI value was measured by changing the oxygen concentration by 0.1%.

The third flammability test was performed using mass loss cone calorimeter in accordance with ISO 13927. Samples with dimensions of 100 × 100 × 10 mm were used for this test. Measurements were carried out using spark ignition and a heat flow rate of 25 kW/m^2^. The distance of the sample from the ignition source was equal 25 mm. The following parameters were measured during the test: time to ignition (TTI), total time of flameout (TTF), percentage mass loss (PLM), heat release rate (HRR), effective heat of combustion (EHC) and total heat release (THR).

#### 3.5.3. Susceptibility to Biodegradation of Polyols Mixture and Foam

The study of susceptibility to biodegradation of polyols mixture and rigid PU foam based on its was carried out in accordance with ISO 17556:2019 using the OxiTop Control S6 apparatus (WTW-Xylem, Rye Brook, NY, USA), which used a respirometric method to measure the oxygen demand necessary for aerobic biodegradation of polymeric materials in soil. The measurement of consumed oxygen was presented using the value of biochemical oxygen demand (BOD), which is the number of milligrams of captured oxygen per mass unit of tested polyurethane material. The OxiTop Control S6 apparatus consisted of six glass bottles with a capacity of 510 mL equipped with rubber quivers and measuring heads, which were used to measure BOD. They allowed to measure the pressure in the range of 500 to 1350 hPa with an accuracy of 1% at a temperature of 5 to 50 °C. The apparatus also included the OC 110 controller (WTW-Xylem, Rye Brook, NY, USA). It was used for communication between the measuring heads, the user, and the Achat OC computer software (WTW-Xylem, Rye Brook, NY, USA), which was used to interpret the obtained measurement results.

Sifted and dried garden soil with a high humus content and physicochemical parameters such as humidity of 5%, pH of 6, grain diameter below 2 mm (collected in Szczepanowo, Kuyavian-Pomeranian Voivodeship, Poland) was used as a biodegradation environment. The measurement was carried out in a system consisting of 200 mg of tested sample (polyol or foam), 200 g of soil and 100 g of distilled water. The hermetic-closed system was placed in a laboratory incubator at 20 ± 0.2 °C and thermostated at this temperature for 28 days.

The biochemical oxygen demand (BOD) for a single OxiTop Control S6 bottle was determined from Equation (4) taking into account the BOD of the tested system reduced by the BOD of the soil and concentration of the tested compound in the soil.

(4) $$BOD_{S}=\frac{BOD_{x}\;-\;BOD_{g}}{c}$$

where *S*—number of measurement days, *BOD_S_*—biochemical oxygen demand of the analyzed sample within *S* days (mg/L), *BOD_x_*—biochemical oxygen demand of the measuring system (bottle with sample and soil) (mg/L), *BOD_g_*—biochemical oxygen demand of soil without a sample (mg/L), *c*—sample concentration in the tested system (mg/L).

The degree of biodegradation of the polyols mixture or foam based on its was determined based on Equation (5):

(5) $$D_{t}=\frac{BOD_{S}}{TOD}\;\times 100\%$$

where *D_t_*—biodegradation degree of sample (%), *TOD*—theoretical oxygen demand (mg/L). 

The theoretical oxygen demand for each system was calculated individually. It was assumed that the carbon contained in the tested samples turns into CO_2_, hydrogen into H_2_O, nitrogen into NH_3_, and silicon into SiO_2_ as a result of the biodegradation process in aerobic conditions. For a compound with a known summary chemical formula containing C, H, N, Si and O elements with mass m, the TOD value can be calculated from the Equation (6):

(6) $$TOD=\frac{16\cdot \;\left[2c\;+\;0.5\cdot \;\left(h\;-3n\right)\;+\;2si-\;o\right]}{m}$$

where: *c*, *h*, *n*, *si*, *o*—mass shares of elements in the macromolecule of biodegradable material (-) and *m*—sample mass of biodegradable material (g).

#### 3.5.4. FTIR Analysis

Foams were tested in FTIR spectroscopy using Nicolet iS20 (Thermo Scientific, Waltham, MA, USA) spectrophotometer in range from 400 to 4000 cm^−1^.

## 4. Conclusions

Two polyol raw materials were synthesized as part of the research. The first polyol was a polyetherol obtained on the basis of metasilicic acid, while the second polyol was the product of the glycolysis reaction of various poly(lactic acid) waste. The obtained polyols were subjected to detailed physicochemical, analytical and spectroscopic tests. It was found, on the basis of the obtained test results, that they were odorless liquids with different densities of 1.188 g/cm^3^ for PLA-based polyol and 1.250 g/cm^3^ for MSA-based polyol and viscosities of 210 mPa·s for PLA-based polyol and 33,442 mPa·s for MSA-based polyol. There were also noted significant differences in the values of the hydroxyl numbers and acid numbers. HNs of polyols were 491 mg KOH/g for eco-polyol and 807 mg KOH/g for polyol based on metasilicic acid, respectively, while ANs of polyols were 2.0 mg KOH/g for eco-polyol and 4.2 mg KOH/g for polyol based on metasilicic acid, respectively. Spectroscopic tests confirmed the assumed chemical structure of the obtained compounds, confirming their suitability for the synthesis of polyurethane materials.

The new polyols were used in the mixture during the development of the optimal formulation of rigid polyurethane foam. The optimal amount of eco-polyol in the mixture was 50 wt.% With respect to the total amount of polyols in the formulation. The optimal amount of eco-polyol in the mixture was 50 wt.% in relation to the total weight of polyol raw materials in the formulation. The increase in the amount of polyol raw material in the formulation (over 50 wt.%) significantly deteriorated the properties of the obtained foams. This was due to the high linearity of the PLA-based polyol structure. The addition of this type of structure into foam matrix was associated with a decrease in cross-linking of the obtained material, and thus a deterioration of mechanical properties and a decrease in foam stiffness. The effect of the amount of catalyst, surfactant, isocyanate raw material and blowing agent was also determined.

The use of equal-mass amounts of PLA-based polyol and MSA-based polyol to obtain rigid polyurethane foam had a positive effect on its properties. A polyurethane foam containing only a polyol based on metasilicic acid in the formulation was used as a reference foam. The obtained foams were tested for physico-mechanical, thermal and thermal insulation properties, as well as flammability. The addition of eco-polyol decreased the burning rate of the foam and significantly improved its thermal resistance due to the presence of the silicon heteroatom. The foam based on a mixture of polyols endured long-term exposure to temperatures of 175 °C, which was its advantage. The use of mixture of eco-polyol based on PLA waste and MSA-based polyol improved most of the properties in comparison with the reference foam, e.g., it decreased the apparent density from 95.5 kg/m^3^ to 89 kg/m^3^, improved the aging resistance and decreased the thermal conductivity to 0.034 W/(m·K). The thermal conductivity coefficient of the reference foam was 0.037 W/(m·K).

The use of PLA-based polyol in the formulation changed the values of water absorption from 3.82, 4.07 and 6.68% for the reference foam to 0.59, 2.74 and 5.14% for the modified foam after 5 min, 3 h and 24 h of immersion, respectively. The lower water absorption and the lower thermal conductivity coefficient of RPUFs are advantageous features due to the possibility of using this material in thermal insulation application. Moreover, the biodegradation tests showed that the polyurethane foam based on polyols mixture was biodegradable, which is of great importance in the case of waste disposal.

The use of a recycling-based polyol raw material in a polyurethane formulation can be an excellent alternative to petrochemical raw materials used in the polyurethane industry. Polyurethane materials based on it can be an excellent proposition for the civil engineering. Moreover, limiting the use of petrochemical raw materials in favor of alternative raw materials fits perfectly into the doctrine of efficient use of resources and the principles of green chemistry. 

## 5. Patents

The synthesis of eco-polyol based on PLA waste was carried out using the method described in the Polish Patent Application No. P.424629.

## Figures and Tables

**Table 1:** Properties of synthesized polyols.

Parameter		PLA-Based Polyol	MSA-Based Polyol
pH (-)		6.0	5.0
Density (g/cm^3^)		1.188	1.250
Viscosity (mPa·s)		210	33442
HN (mg KOH/g)		491	807
AN (mg KOH/g)		2.0	4.2
Elemental analysis	C (%)	44.36	46.34 *
	H (%)	7.62	8.38 *
	O (%)	48.02	42.96 *
	Si (%)	0.00	2.32 *
Color (-)		light-yellow	brown
Smell (-)		no smell	no smell
Number-average molecular weight (g/mol)		309	314
Weight-average molecular weight (g/mol)		351	899
Polydispersity (-)		1.14	2.86

* Elemental analysis was performed on a product devoid of poly(silicic acid).

**Table 2:** Processing times of the obtained RPUFs and their macroscopic characteristics.

Foam Symbol	Cream Time (s)	Free Rise Time (s)	Tack Free Time (s)	Comments
Ref.	30 ± 1	55 ± 1	56 ± 1	rigid; regular pores;
1	30 ± 1	50 ± 1	50 ± 1	large, irregular pores;
2	25 ± 1	50 ± 1	60 ± 1	non-gelled surface;
3	23 ± 1	43 ± 1	43 ± 1	cracked surface;
4	30 ± 1	58 ± 1	58 ± 1	slight cracks in the foam;
5	35 ± 1	60 ± 1	60 ± 1	rigid; regular pores;
6	40 ± 1	75 ± 1	75 ± 1	rigid; regular pores;

**Table 3:** Results of statistical analysis of SEM micrographs.

Foam Symbol	Pore Size (μm)	Thickness of Pore Wall (μm)	Content of Pores Per Area Unit (Pores/mm^2^)
Ref.	144 ± 52	14 ± 3	18 ± 4
6	162 ± 66	15 ± 2	20 ± 5

**Table 4:** The results of physico-mechanical properties of RPUFs.

Foam Symbol	Apparent Density (kg/m^3^)	Compressive Strength (kPa)	Water Absorption (%)		
			after 5 min	after 3 h	after 24 h
Ref.	95.5 ± 1.8	486 ± 10	3.82 ± 0.06	4.07 ± 0.15	6.68 ± 0.26
6	89.3 ± 1.6	493 ± 12	0.59 ± 0.05	2.74 ± 0.12	5.14 ± 0.17

**Table 5:** Results of thermogravimetric analysis.

Foam Symbol	T_5%_ (°C)	T_10%_ (°C)	T_25%_ (°C)	T_50%_ (°C)	T_max_ (°C)	T_g_ (°C)
Ref.	167	221	265	361	220, 340	>200
6	230	270	325	370	270, 340, 390	111

**Table 6:** Comparison of the fire properties of the reference foam and the foam obtained from a mixture of polyols before and after temperature exposure.

Type of Foam	Foam Symbol	Mass Loss after 30-day Exposure (%)	Compressive Strength (kPa)	LOI (%)	Length of Burnt Foam (mm)	Burning Rate (mm/s)	Mass Loss after Burning (%)	Comments
Non-thermostated	Ref.	-	486 ± 10	21.1	150	2.03	100	melts, drips, sparks visible;
	6	-	493 ± 12	20.9	150	1.37	49.6	-
Thermostated at 150 °C	Ref.	24.00	1275 ± 24	24.4	105	2.10	2.45	burns in the flame, goes out after removing flame;
	6	10.44	1296 ± 32	23.9	4	-	0.5	burns in the flame, goes out after removing flame;
Thermostated at. 175 °C	Ref.	36.5	5051 ± 35	36.0	0	0	0.47	does not burn in the flame;
	6	26.01	2611 ± 42	33.2	0	0	0.5	does not burn in the flame;

**Table 7:** The test results of RPUFs using the cone calorimeter.

Foam Symbol	TTI (s)	TTF (s)	PML (%)	HRR (kW/m^2^)	THR (MJ/m^2^)	EHC (MJ/kg)
**Ref.**	7	75	65.6	65.6	4.7	53.7
**6.**	26	159	74.5	132.9	9.7	44.5

**Table 8:** Mass shares of individual elements in foams.

Element	C	H	O	N	Si
**Ref.**	0.5961	0.0660	0.2447	0.0707	0.0225
**6**	0.6247	0.0548	0.2403	0.0697	0.0105

**Table 9:** Results of susceptibility to biodegradation of foams.

Foam Symbol	BOD_28_ (mg/L)	TOD (mg/L)	D_t_ (%)
**Ref.**	0.00	73.56	0.00
**6**	54.90	80.25	68.41

**Table 10:** Formulations of foams based on the mixture of MSA- and PLA-polyols.

Formulation Number	Amount of Component (g)					
	MSA-Based Polyol	PLA-Based Polyol	pMDI	Water	TEA	Silicon L-6900
Ref.	100	0	132	2	1.6	2.3
1	50	50	180	3	1.6	2.3
2	50	50	180	2	1.6	2.3
3	50	50	170	2	1.6	2.3
4	50	50	170	2	1.1	2.3
5	50	50	170	2	0.7	2.3
6	50	50	170	2	0.4	1.9

## Data Availability

Data is contained within the article or supplementary material.

## Supplementary Materials

The following are available online at https://www.mdpi.com/1422-0067/22/1/69/s1. Figure S1. GPC chromatogramof eco-polyol based on PLA waste. Table S1. Side products of EC reaction used for calibration of gas chromatography. Table S2. Interpretation of MALDI-ToF spectrum of oligoetherol obtained from MSA. Table S3. Amount of side products [wt. %] in obtained oligoetherol from MSA. Table S4. Results of GPC chromatography analysis.
