# Supplementary material for: Use of a Mixture of Polyols Based on Metasilicic Acid and Recycled PLA for Synthesis of Rigid Polyurethane Foams Susceptible to Biodegradation
Source: Int J Mol Sci. 2020 Dec 23;22(1):69. doi: 10.3390/ijms22010069 (PMC7795053; doi:10.3390/ijms22010069)
Supplement: Supplementary file 1 [file ijms-22-00069-s001.pdf]

Supplementary files

Use of a mixture of polyols based on metasilicic acid and recycled PLA for synthesis of rigid polyurethane foams susceptible to biodegradation

Joanna Paciorek-Sadowska [1,*], Marcin Borowicz [1,*], Ewelina Chmiel [2] and Jacek Lubczak [2]

[1] Department of Polyurethane Chemistry and Technology, Institute of Materials Engineering, Kazimierz Wielki University, Chodkiewicza 30 St, 85-064 Bydgoszcz, Poland;

[2] Department of Organic Chemistry, Rzeszów University of Technology, Al. Powstańców Warszawy 6, 35-959 Rzeszów, Poland; jml@prz.edu.pl (J.L.); ewelinachmiel@prz.edu.pl (E.C.);

* Correspondence: sadowska@ukw.edu.pl (J.P.-S.); m.borowicz@ukw.edu.pl (M.B.); Tel.: +48-52-34-19-286

1. Analysis of GPC and MALDI ToF of MSA-polyol

MALDI ToF spectra (Matrix-Assiated Laser Desorption Ionization Time of Flight) of oligoetherols were obtained on Voyager-Elite Perceptive Biosystems (US) mass spectrometer working at linear mode with delayed ion extraction, equipped with nitrogen laser working at 352 nm. The method of laser desorption from matrix gold was used. Therefore the observed peaks corresponded to the molecular ions plus Au and K^+ (from catalyst) ions. The samples with diluted with water to 0.5 mg/cm^3.

Molecular mass (number-averaged and weight-averaged) and polydispersity of oligoetherol was determined by gel permeation chromatography using the following parameters: 25 ± 0.1°C temperature, 1 cm^3 / min volume flow of eluent, 20 μdm^3, volume of inlet chamber, 4 - 5 mg / cm^3 polymer concentration, 30 minutes analysis time, eluent: N,N-dimethylformamide, calibration reference: polystyrene.

In order to identify side products (glycols and polyglycols) formed in the reaction of semi-product MSA : GL = 1 : 4 with EC and the compounds formed in consecutive reactions with EC, the oligoetherols were separated chromatographically using cyklohexanone (analytical grade S.A. POCH, Gliwice, Poland) as internal standard. The gas chromatograph HP 4890A was used, equipped with HP-FFAP column of 30 m length, 0,53 mm diameter, 1,5 μm film thickness and 220 °C port temperature and temperature profile: 50 – 220 ºC, with 20 deg/min heating rate, the helium flow 18,3 cm^3/min, and 0,2 μdm^3 sample volume. Series of reference substances were used: ethylene glycol (MEG), diethylene glycol (DEG), triethylene glycol (TEG) tetraethylene glycol (TeEG), (pure Aldrich, UK). The percentage of diols in products was determined based on calibration curves with the same internal standard using equation (S1):

$$\frac{S_{cd}}{S_t} = a \times \left(\frac{m_{cd}}{m_t}\right) + b \qquad \text{(S1)}$$

where: m_{cd}, m_t – diol mass or consecutive product of its reaction with alkylene carbonate and mass of standard, respectively,

S_{cd}, S_t – integrated peak area of diol or consecutive product and standard, respectively,

a, b – experimental coefficients of calibration curves.

The calibration coefficients and retention times of diols are collected in Table S1. Mass of products obtained from EC and water and mass of products of consecutive reactions bet-ween diols and alkylene carbonates were calculated from formula (1) (m_{cd}). The percentage of side products were calculated considering total sample mass (m_p) according to the equation S2:

$$p_{cd} = \left(\frac{m_{cd}}{m_p}\right) \times 100\% \qquad \text{(S2)}$$

Table S1. Side products of EC reaction used for calibration of gas chromatography.

Substance	Retention time [min]	Coefficients from equation (1)		Correlation coefficient
		a	b	
cyclohexanone	8.53	-	-	
MEG	13.64	2.7939	0.1006	0.9982
DEG	17.47	2.4917	0.1694	0.9988
TEG	20.83	2.6634	0.2392	0.9987
TeEG	25.95	4.4556	0.2740	0.9993

The composition of oligoetherols can be determined by MALDI-ToF spectrometry method (Table S2). The spectra showed trace amounts of substrates identified by the appropriate low M/z, and also corresponding to oligomeric forms of MSA of the general formula $(H_2SiO_3)_n$, where n = 2, 3 or 4 From detailed analysis of MALDI-ToF spectra it can be concluded that even at the appropriate molar ratio of reagents the mixtures are obtained, in which MSA is hydroxyalkylated with GL at various level and the product of reaction between the semiproduct and EC at various level of hydroxyalkylation. Thus, the satellite peaks with masses increased by M/z = 74 or by masses of attached ions from matrix, and also the peaks of masses diminished by M/z =18 due to elimination of water in self-condensation of MSA are present The series of peaks differing by M/z = 44 derived from consecutive addition of EC are also present.

Table S2. Interpretation of MALDI-ToF spectrum of oligoetherol obtained from MSA

Entry	Signal position m/z	Relative inten-sity of signal [%]	The molecular ion structure	Calc. molecular weight [g/mol]
1.	75.145	12.5	GL + H^+	75.087
2.	87.134	5.6	EC	88.062
3.	89.145	2.5	EC + H^+	89.070
4.	97.034	3.8	MSA + H_2O + H^+	97.122
5.	101.143	9.6	MSA + Na^+	101.089
6.	124.981	26.1	EC+ 2 H_2O	124.092
7.	129.142	50.0	2GL - H_2O	130.143
8.	133.001	2.4	MSA + GL - H_2O	134.163
9.	140.954	42.9	GL + OE + Na^+	141.122
10.	145.117	63.4	GL + 2OE – H_2O	144.170
11.	159.146	44.4	GL + OE + K^+	157.230
12.	173.147	55.4	MSA + GL - H_2O + K^+	173.261
13.	189.130	80.4	2GL + K^+	187.257
14.	203.144	68.0	3GL - H_2O	204.222
15.	219.129	65.3	MSA + GL + OE + Na^+	219.221
16.	233.146	83.3	2 MSA – 2H_2O + GL + K^+	233.345
17.	249.144	50.6	2 MSA + GL + H_2O + H^+	249.300
18.	263.149	56.3	MSA + 2GL + K^+	265.355
19.	277.167	66.0	MSA + 2GL – H_2O + OE + Na^+	275.285
20.	279.127	21.5	4GL - H_2O + H^+	279.309
21.	289.168	58.7	2 MSA – H_2O + 2GL - H_2O + Na^+	291.316
22.	307.172	100.0	MSA + 2GL + OE + K^+	309.408
23.	323.154	57.5	2 MSA – H_2O + 2GL + K^+	325.439
24.	333.187	17.1	4 MSA - 3H_2O + GL + H^+	333.438
25.	337.177	49.2	2 MSA - H_2O + Au + H^+	335.960
26.	351.191	71.0	5GL - H_2O	352.380

			MSA + GL + Au + H^+	350.153
27.	363.193	51.4	2 MSA + GL + 3OE + H^+	363.444
28.	381.200	88.1	MSA + 2GL + 3OE + Na^+	381.406
29.	393.934	31.5	MSA + GL + OE + Au	393.198
30.	397.187	43.5	3 MSA - $2H_2O$ + Au + H^+	396.242
31.	407.214	17.6	MSA + 2GL - H_2O + Au + H^+	406.217
32.	411.205	41.8	2 MSA + 2GL + 2OE + H_2O + H^+	411.485
33.	425.220	66.3	2 MSA + 3GL + OE + H^+	423.496
34.	437.222	38.3	MSA + GL + 2OE + Au	437.251
35.	453.200	27.4	2 MSA - H_2O + GL + OE + Au	453.282
36.	455.229	69.4	4 MSA - $3H_2O$ + Au	455.318
37.	467.228	22.5	MSA + 2GL + OE + Au	467.277
38.	469.229	17.3	3 MSA - $2H_2O$ + GL + Au	469.313
39.	485.230	33.5	2 MSA - H_2O + 2GL + Au + H^+	484.316
40.	499.251	52.9	7GL - H_2O<br>2 MSA - H_2O + GL + 2OE + Au +H^+	500.538<br>498.343
41.	511.249	27.1	MSA + 2GL + 2OE + Au	511.330
42.	527.525	18.5	2 MSA - H_2O + 2GL + OE + Au	527.361
43.	529.256	51.0	4 MSA - $3H_2O$ + GL + Au	529.397
44.	541.257	15.4	2 MSA - H_2O + GL + 3OE + Au	541.388
45.	543.270	18.2	MSA + 3GL + OE + Au + H^+	542.364
46.	559.259	23.2	2 MSA - H_2O + 3GL + Au + H^+	558.395
47.	573.277	40.4	4 MSA - $3H_2O$ + GL + OE + Au	573.450
48.	585.279	18.5	MSA + 3GL + 2OE + Au<br>2 MSA - H_2O + GL + 4OE + Au	585.409<br>585.441
49.	590.900	39.5	2 MSA + 2GL + 2OE +Au	589.429
50.	603.287	34.9	4 MSA - $3H_2O$ + 2GL + Au	603.476
51.	619.267	13.1	3 MSA - $2H_2O$ + 3GL + Au + H^+<br>4 MSA - $3H_2O$ + GL + 2OE + Au + H^+	618.479<br>618.551
52.	633.287	15.4	3 MSA - $2H_2O$ + 2GL + 2OE + Au +H^+	632.506
53.	647.309	28.7	4 MSA - $3H_2O$ + 2GL + OE + Au	647.529
54.	659.290	12.1	MSA + 4GL + 2OE + Au	659.488
55.	663.288	11.0	3 MSA - $2H_2O$ + 3GL + OE + Au + H^+	662.532
56.	677.318	20.5	4KK - $3H_2O$ + 3GL + Au	677.555
57.	693.297	6.8	3 MSA - $2H_2O$ + 4GL + Au + H^+<br>4 MSA - $3H_2O$ + 2GL + 2OE + Au + H^+	692.558<br>692.590
58.	707.315	9.6	3 MSA - $2H_2O$ + 3GL + 2OE + Au +H^+<br>2 MSA - H_2O + 5GL + Au + H^+	706.585<br>706.553
59.	721.340	19.8	MSA + 6GL + Au + H^+	720.548
60.	737.315	7.5	4 MSA - $3H_2O$ + 2GL + 3OE + Au + H^+<br>3 MSA - $2H_2O$ + 4GL + OE + Au + H^+	736.643<br>736.611
61.	751.350	12.1	4 MSA - $3H_2O$ + 4GL + Au	751.634
62.	781.341	5.3	2 MSA - H_2O + 6GL + Au + H^+	780.632
63.	795.368	11.4	4 MSA - $3H_2O$ + 4GL + OE + Au	795.687
64.	825.357	7.1	4 MSA - $3H_2O$ + 5GL + Au	825.713
65.	839.406	4.8	4 MSA - $3H_2O$ + 4GL + 2OE + Au	839.740
66.	869.414	6.9	MSA + 8GL + Au + H^+	868.706

MSA, *GL and EO – the structural fragments formed upon reaction of MSA with GL and EC, respectively.*

The obtained oligoetherols were analyzed by gas chromatography in order to determine the amount of side products formed in reaction of EC with water released from products of MSA hydroxyalkylation or with diols. In separate control experiments the products of EC with reference diols

like glycol and polyglycols were analyzed chromatographically (Table S1). It has been found that the percentage of glycols and their consecutive products has not exceeded 20 mass % in the products (Table S3). The presence of low molecular products in oligoetherol corroborate well with hydroxyl number (Table S2) which is 807 mg KOH/g, The number-averaged molecular mass was determined by GPC chromatography as 314 g/mol, due to the presence of low molecular weight admixtures. Weight-averaged molecular mass was 899 g/mol with polydispersity degree 2.86. The result on M_n and M_w are in good accordance with the masses calculated from assumed oligoetherol and side products (Table S3), which were Mn = 333 g/mol and Mw = 917 g/mol, respectively.

Table S3. Amount of side products [wt. %] in obtained oligoetherol from MSA

ED	DEG	TEG	TeEG	∑
7.9	9.0	2.4	0.6	19.9

2. GPC analysis of PLA-polyol

The GPC chromatogram of eco-polyol based on waste PLA is shown in Figure S1. The interpretation of the obtained chromatogram is presented in Table S4.

Figure S1. GPC chromatogram of eco-polyol based on PLA waste

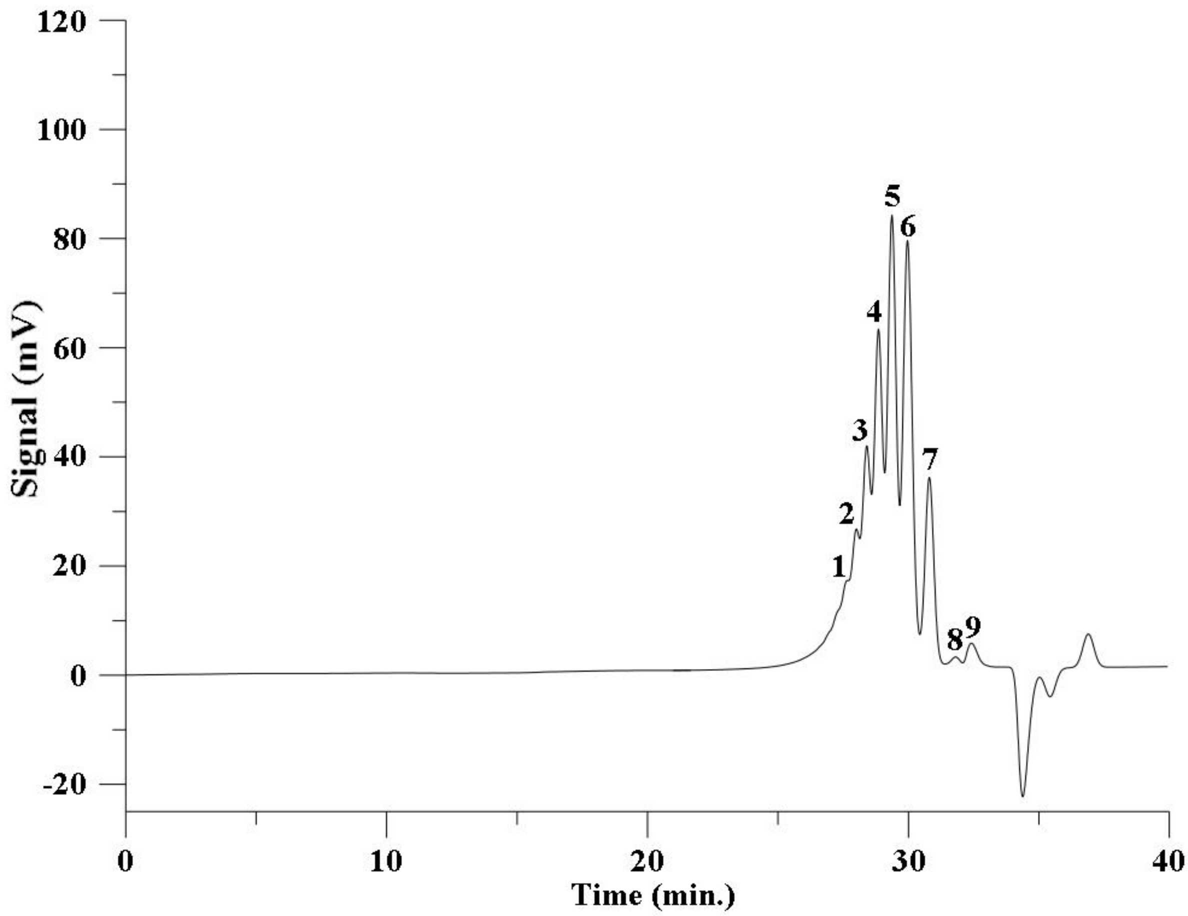

Table S4. Results of GPC chromatography analysis

Number of peak	M_n (g/mol)	M_w (g/mol)	D (-)
**1.**	647	671	1.04
**2**	494	494	1.00
**3**	431	431	1.00
**4**	372	373	1.0.
**5**	315	315	1.00
**6**	259	259	1.00
**7**	198	199	1.01
**8**	146	146	1.00
**9**	116	117	1.01
**Average weight**	**309**	**351**	**1.14**
